# Electrical Characterizations of Planar Ga_2_O_3_ Schottky Barrier Diodes

**DOI:** 10.3390/mi12030259

**Published:** 2021-03-03

**Authors:** Shiyu Zhang, Zeng Liu, Yuanyuan Liu, Yusong Zhi, Peigang Li, Zhenping Wu, Weihua Tang

**Affiliations:** 1 Laboratory of Information Functional Materials and Devices, School of Science & State Key Laboratory of Information Photonics and Optical Communications, Beijing University of Posts and Telecommunications, Beijing 100876, China; zhangshiyu626@163.com (S.Z.); zengliu@bupt.edu.cn (Z.L.); yusongzhi@bupt.edu.cn (Y.Z.); zhenpingwu@bupt.edu.cn (Z.W.); 2 College of Electronic and Optical Engineering & College of Microelectronics, Nanjing University of Posts and Telecommunications, Nanjing 210023, China; 3 National and Local Joint Engineering Laboratory for RF Integration and Micro-Packing Technologies, Nanjing University of Posts and Telecommunications, Nanjing 210023, China; 4 Center of Materials Science and Optoelectronics Engineering, University of Chinese Academy of Sciences, Beijing 100049, China; liuyy@semi.ac.cn; 5 The Engineering Research Center for Semiconductor Integrated Technology, Institute of Semiconductors, Chinese Academy of Sciences, Beijing 100083, China

**Keywords:** Ga_2_O_3_, Schottky diode, I–V, C–V

## Abstract

In this work, a Schottky barrier diode (SBD) is fabricated and demonstrated based on the edge-defined film-fed grown (EFG) Ga_2_O_3_ crystal substrate. At the current stage, for high resistance un-doped Ga_2_O_3_ films and/or bulk substrates, the carrier concentration (and other electrical parameters) is difficult to be obtained by using the conventional Hall measurement. Therefore, we extracted the electrical parameters such as on-state resistance (*R_on_*), Schottky barrier height ($\phi_{B}$), the ideal factor (*n*), series resistance (*R_s_*) and the carrier concentration (*N_d_*) by analyzing the current density–voltage (J–V) and capacitance–voltage (C–V) curves of the Ga_2_O_3_-based SBD, systematically. The detailed measurements and theoretical analysis are displayed in this paper.

## 1. Introduction

Owing to the suitable energy band gap (E_g_) of ~4.9 eV, a high Baliga’s figure of merit (FOM) of more than 3200, a high Huang’s material FOM of 279, Johnson’s FOM of 2844, and a high theoretical breakdown field (E_BR_) of ~8 MV/cm, gallium oxide (Ga_2_O_3_) has been widely employed to construct optical detectors and power electronics along with low direct current losses and stable operations [1,2,3]. Till now, Ga_2_O_3_-based Schottky barrier diodes (SBDs) have achieved high breakdown voltages of 2300 V in a vertical edge-terminated SBD [4] and 3000 V in a lateral field-plated (FP) SBD [5], suggesting strong competitiveness superior to GaN and SiC. Owing to the low dark current and fast response speed [6], the Ga_2_O_3_-based Schottky diode photodetectors also have drawn much attention for high performances. For instance, the high photo responsivity (R), high external quantum efficiency (EQE), large specific detectivity (D*), and short response time in Ni/β-Ga_2_O_3_ [7], Pt/ε-Ga_2_O_3_ [8], graphene/β-Ga_2_O_3_ [9], and MXenes/β-Ga_2_O_3_ [10] photodiodes are realized.

In electronic devices, the electrical parameters, such as carrier concentration, are key issues that affect the device design, construction, and operation. For doped Ga_2_O_3_ and/or the flakes exfoliated from the bulk single crystal, the electron concentrations are reported as 10^16^–10^20^ cm^−3^ [11,12,13,14], while in fact, the un-doped Ga_2_O_3_ has a high resistance due to the large E_g_, thus it is very hard to obtain some electrical parameters via the conventional Hall measurement. Additionally, a systematic and comprehensive study for electrical characterizations such as on-state resistance (*R_on_*), Schottky barrier height ($\phi_{B}$), the ideal factor (*n*), series resistance (*R_s_*) and the carrier concentration (*N_d_*) of Ga_2_O_3_ Schottky devices is less reported. Therefore, to solve this problem, we fabricated a Ga_2_O_3_-based SBD, and then performed the electrical characterizations through the J–V, H(J)–J, and C–V characteristic curves.

## 2. Experimental Section

**Figure 1:**
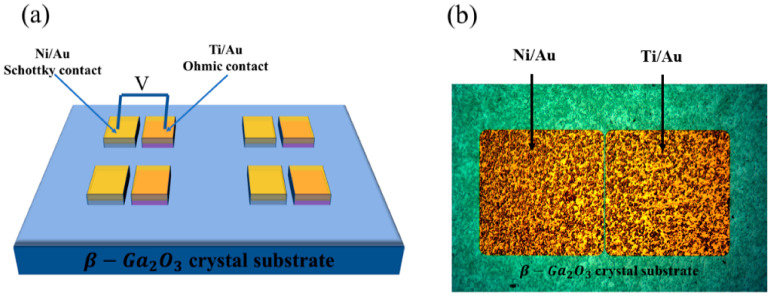
(**a**) The geometry of the Schottky diodes with $\mathrm{Ni}/\beta -{\mathrm{Ga}}_{2}O_{3}/\mathrm{Ti}$ structure. (**b**) Optical microscopy image of the fabricated Schottky diode.

The Ga_2_O_3_ crystal substrate was grown by the edge-defined film-fed grown method, whose scale is 10 mm × 10 mm. For fabricating the Schottky diode, photolithography, lift-off, and electron beam vapor techniques were used to finish the Ni/Au (Schottky, 30 nm/100 nm) and Ti/Au (Ohmic, 30 nm/100 nm) electrode patterns as shown in Figure 1b. X-ray diffraction (XRD), X-ray photoelectron spectroscopy (XPS), and scanning electron microscopy (SEM) were performed to verify the quality of the Ga_2_O_3_ crystal substrate. The electrical test was executed with Keithley 4200 analysis equipment in the air at room temperature. The area of the electrode pattern is 2.4 × 10^−3^ cm^2^. Figure 1a is the schematic of the planar Ga_2_O_3_ SBDs.

## 3. Results and Discussion

**Figure 2:**
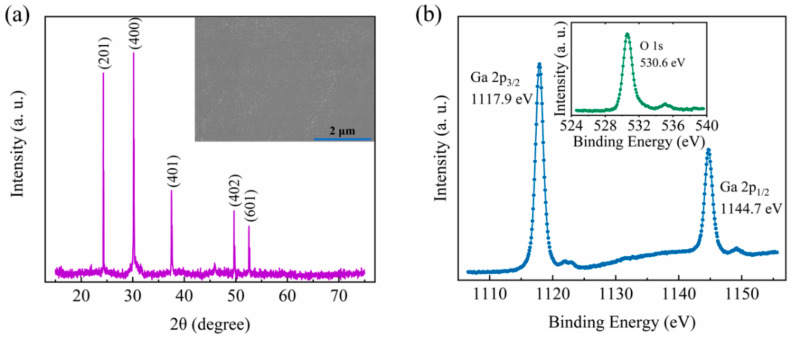
(**a**) The X-ray diffraction (XRD) pattern and (**b**) the X-ray photoelectron spectroscopy (XPS) of the Ga_2_O_3_ substrate, of which the scanning electron microscope (SEM) image is shown in (**a**) inset.

As shown in Figure 2a, the XRD pattern of the edge-defined film-fed (EFG)-grown Ga_2_O_3_ substrate is displayed, showing (201), (400), (401), (402), and (601) orientations. The prepared Ga_2_O_3_ crystal is polycrystalline β-Ga_2_O_3_. The SEM image of the surface of the Ga_2_O_3_ substrate is portrayed in Figure 2a inset, suggesting a decent crystallization with well-defined boundaries and uniform claviform grains. In Figure 2b, the binding energies of Ga 2p_1/2_, Ga 2p_3/2_, and O 1s are determined to be 1144.7 eV, 1117.9 eV, and 530.6 eV by XPS, they are similar to the reported values as shown in Table 1, verifying the formation of the Ga_2_O_3_ semiconductor.

**Figure 3:**
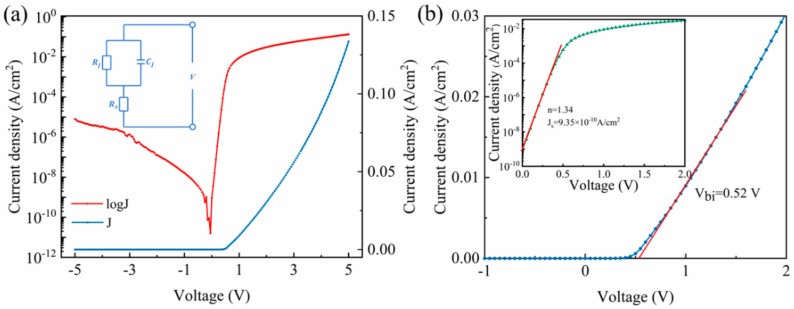
(**a**) The current density–voltage (J–V) curves of the Ni/Ga_2_O_3_/Ti Schottky diode, inset is the equivalent circuit diagram, (**b**) the *n*, *J_s_*, and *V_bi_* based on thermionic emission (TE) model.

In Figure 3a, current density–voltage (J–V) curves of the Ni/Ga_2_O_3_/Ti Schottky diode are shown in linear-scale and semi-log scale, respectively. The output current density is 3.04 × 10^−2^ A/cm^2^ and 1.25 × 10^−7^ A/cm^2^ at 2 V and −2 V, respectively, translating to a high rectifying ratio of 2.44 × 10^5^; the fabricated device is on-state at forward voltages while off-state at reverse voltages. By extrapolating the linear region of the log-scaled J–V curve in Figure 3a (red line) to obtain its slope, the on-state resistance (R_on_) is estimated to be 51.5 Ω cm^2^. Furtherly, we use the thermionic emission (TE) model to describe the Ni/Ga_2_O_3_/Ti Schottky diode [18]:

(1) $$J=J_{s}\left(e^{\frac{qV}{nkT}}-1\right)$$

(2) $$J_{s}=A^{*}T^{2}e^{-\frac{\phi_{B}}{kT}}$$

(3) $$\phi_{B}=\frac{kT}{q}\ln\left(\frac{A^{*}T^{2}}{J_{s}}\right)$$

and

(4) $$A^{*}=\frac{qm^{*}k^{2}}{2\pi^{2}\hbar^{3}}$$

where q is the electron charge, *V* is the applied voltage, $J_{s}$ is the reverse saturation current, *n* is the ideal factor, *k* is the Boltzmann constant, *T* is the thermodynamic temperature, $\phi_{B}$ is the Schottky barrier height, and $A^{*}$ is the Richardson constant. For Ga_2_O_3_, its effective electron mass $m^{*}=0.342\;m_{0}$, with $m_{0}$ the mass of the free electron, thus the $A^{*}$ of Ga_2_O_3_ is calculated to be 41.07 A/cm^2^ K^2^ [19].

**Figure 4:**
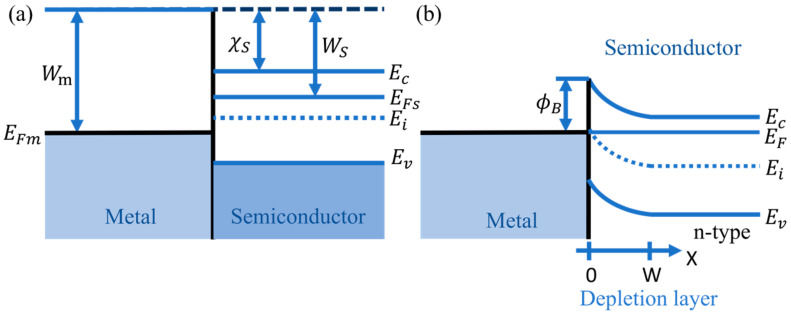
The energy band diagram of the Ni/Ga_2_O_3_/Ti Schottky diode (**a**) before and (**b**) after contact.

As displayed in Figure 3b, with the TE model described in Equations (1)–(4), the *n*, $\phi_{B}$ and turn-on voltage (*V_on_*) are determined to be 1.34, 0.93 eV, and 0.52 V, respectively. In Figure 3b inset, the $J_{s}$ of the Schottky diode is 9.35 × 10^−10^ A/cm^2^. The *n* value between 1 and 2, nearer to 1, indicates a decent Schottky electrical behavior in the fabricated device. The *V_on_* suggests a built-in potential difference (*V_bi_*) of 0.52 eV. In fact, the work function of Ni (*W_m_*) is 5.15 eV [20], and the electron affinity of Ga_2_O_3_ ($\chi_{s}$) is 4.00 eV [21]. The Ni-Ga_2_O_3_ energy band diagram before they make contact is shown in Figure 4a. According to the Schottky–Mott rule [22,23], the Ni-Ga_2_O_3_ interface barrier is about 1.15 eV, i.e., the difference between *W_m_* and $\chi_{s}$. After they make contact, as given Figure 4b, the conduction and valence energy band edge (*E_c_* and *E_v_*) bend up, owing to the electron transferring to the electrode. The formation of the $\phi_{B}$ indicates a barrier for electron transport in the device, thus the device has a *V_on_* and rectifying effect.

For the device, the series resistance (*R_s_*) is a key parameter affecting the performances of the Schottky diode, modeled by a combination of a capacitance (*C_j_*) and a resistor (*R_j_*) as shown in Figure 2a inset. When the current flow is in the diode, it has a relationship with voltages applied on the device [24]:

(5) $$V_{D}=V-JAR_{s}$$

where $V_{D}$ is the voltage on the two sides of the diode, *V* is the applied voltage, and *A* is the area of the electrode. Other than the homojunction device, in the Schottky junction similar to the heterojunction, the key role in determining the electrical behavior could not be told by diffusion current model. When $V_{D}>3kT/e$, the current in diode could be described by the thermionic electron model:

(6) $$J=J_{s}\exp\left[e\left(V-JAR_{s}\right)/nkT\right]$$

**Figure 5:**
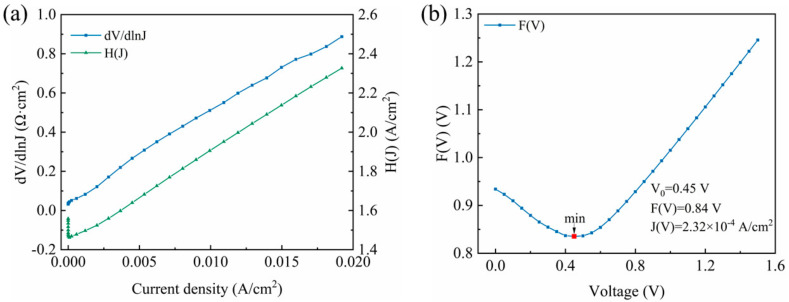
(**a**) The (dV/dlnJ)- J and H(J)-J curve, (**b**) F(V)-V curves of the Schottky diode.

Thus,

(7) $$V=AR_{s}J+n\phi_{B}+\frac{nkT}{e}\ln\left(\frac{J}{A^{*}T^{2}}\right)$$

(8) $$dV/dlnJ=AR_{s}J+\frac{nkT}{e}$$

moreover, H(J) is defined as

(9) $$H\left(J\right)=V-\frac{e}{nkT}\ln\left(\frac{J}{A^{*}T^{2}}\right)=AR_{s}J+n\phi_{B}$$

based on Equation (8), through the linear fitting (dV/dlnJ)-J curve shown in Figure 5a (blue dot-line curve), the *n* and *R_s_* of the Schottky diode are estimated to be 1.67 and 45.5 Ω cm^2^. Then, according to the Equation (9), $\phi_{B}$ and *R_s_* of the Schottky diode could be obtained as 0.86 eV and 46.6 Ω cm^2^, by fitting the H(J)-J curve displayed in Figure 5a (green dot-line curve). In addition, according to the energy band diagram in Figure 3, $\phi_{B}$ can be demonstrated as $\phi_{B}=eV_{bi}+\left(E_{c}-E_{f}\right)$ [18], where $E_{c}$ and $E_{f}$ are the conduction minimum and Fermi level of the Ga_2_O_3_. $E_{c}-E_{f}=kTln\left(\frac{N_{c}}{N_{d}-N_{a}}\right)$, where $N_{c}$ is effective state density at conduction band, expressed as $N_{c}=2{\frac{2\pi m^{*}kT}{h^{2}}}^{3/2}$, thus the electron concentration of the Ga_2_O_3_ can be calculated to be 1.04 × 10^19^ cm^−3^ [25]. What’s more, based on the Norde’s method [26], a function F(V) is defined as:

(10) $$F\left(V\right)=\frac{V}{2}-\frac{kT}{e}\ln\left(\frac{J\left(V\right)}{A^{*}T^{2}}\right)$$

so, $\phi_{B}$ and *R_s_* could be expressed as:

(11) $$\phi_{B}=F\left(V_{0}\right)+\frac{V_{0}}{2}-\frac{kT}{e}$$

and

(12) $$R_{s}=\frac{kT}{eJ\left(V_{0}\right)A}$$

where $V_{0}$ is the voltage at the rock bottom of this function, $F\left(V_{0}\right)$ and $J\left(V_{0}\right)$ are the corresponding functions of $V_{0}$. As shown in Figure 5b, the $V_{0}$ has been marked as 0.45 V. On the basis of Equations (10)–(12), $\phi_{B}$ and *R_s_* of the Schottky diode could be obtained as 1.04 eV and 112.1 Ω cm^2^.

The Schottky barrier region in a Schottky diode could be regarded as a planar capacitance, which can be shown as $C=\frac{\varepsilon_{s}\varepsilon_{0}A}{W}$ [25,27], where *W* is the width of the depletion, $\varepsilon_{s}$ and $\varepsilon_{0}$ are the permittivity of Ga_2_O_3_ ($\varepsilon_{s}$~10) and vacuum permittivity (8.85 × 10^−12^ F/m). Based on Poisson’s equation, it can be described as:

(13) $$\frac{1}{C^{2}}=\frac{2}{qN_{d}\varepsilon_{s}\varepsilon_{0}A^{2}}\left(V_{bi}-V-\frac{kT}{q}\right)$$

**Figure 6:**
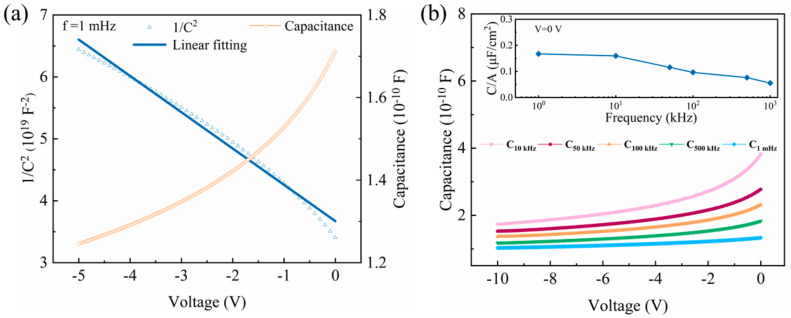
(**a**) The C–V and $\frac{1}{C^{2}}$-V curves, (**b**) the C–V curves with different frequency.

As shown in Figure 6a, the capacitance–voltage (C–V) and $\frac{1}{C^{2}}$-V curves are displayed with a frequency (f) of 100 kHz, and it can be clearly seen that the C decreases from 1.75 × 10^−10^ F to 1.27 × 10^−10^ F, with a gradual slowing rate. With Equation (13), the *N_d_* could be fitted through the $\frac{1}{C^{2}}$-V curve to be 4.36 × 10^17^ cm^−3^. In addition to Figure 6b, the C is almost unchanged with different frequencies, due to the fact that the current in a Schottky diode is caused by the entrance of a majority carrier into the metal side instead of the charge accumulation, i.e., there is no storage effect, thus no diffusion capacitance. Such characterization is beneficial in constructing high-frequency devices for Ga_2_O_3_.

Table 2 lists and compares the basic electrical parameters of some Ga_2_O_3_ SBDs in the past two years. From the table, we comprehensively discussed and calculated the basic electrical parameters by different analysis methods of J–V and C–V.

## 4. Conclusions

In this work, an EFG-grown Ni/Ga_2_O_3_/Ti Schottky diode is fabricated and characterized. By different analysis methods of J–V and C–V, its electrical parameters such as on-state resistance (*R_on_*), Schottky barrier height ($\phi_{B}$), the ideal factor (*n*), series resistance (*R_s_*) and the carrier concentration (N_d_) are obtained and discussed, systematically and comprehensively. The further investigation of Ga_2_O_3_ materials and devices is in inherent demand due to its excellent properties and the prospect of applications.

## Figures and Tables

**Table 1:** Comparison of the binding energies of Ga 2p_1/2_, Ga 2p_3/2_, and O 1s.

Ga 2p_1/2_ (eV)	Ga 2p_3/2_ (eV)	O 1s (eV)	Reference
1144.7	1117.9	530.6	This work
1145.18	1118.28	531.18	[15]
1145	1120	530.7	[16]
1145.5	1118.5	531.4	[17]

**Table 2:** Basic performance parameters of some reported Ga_2_O_3_ Schottky diodes in 2019 and 2020.

Substrate	Anode Metal	$V_{br}\;(V)$	$\frac{I_{on}}{I_{off}}$	*n*	$qV_{bi}\;(\mathrm{eV})$	$q\phi_{B}\;(\mathrm{eV})$	$N_{d}\;({\mathrm{cm}}^{-3})$	$R_{on}$ $\left(m\Omega \cdot cm^{2}\right)$	$R_{s}$ $\left(m\Omega \cdot cm^{2}\right)$	Ref
Wafer (100)	Ni	-	2.44 × 10^5^	1.34	0.52	0.93	$4.36\times 10^{17}$	51.5	45.5	This work
Epi layer (001)	Ni	261	-	1.21	0.74	-	$1.77\times 10^{16}$	77.3	-	[27]
Wafer $\left(\bar{2}01\right)$	Ni	23	$2\times 10^{11}$	1.21		1.31–1.64	$1.96\times 10^{18}$	1.54	-	[28]
Film (100)	Mo	260	-	-	-	1.55	$2\times 10^{17}$	-	-	[29]
Wafer $\left(\bar{2}01\right)$	Ni/Pt	-	-	1.14	1.3	1.37	$1.45\times 10^{18}$	-	-	[30]
Epi layer (001)	Ni	-	$1\times 10^{10}$	1.17	-	1.02	$6.9\times 10^{15}$	40	-	[31]
